# Low-Level Prenatal and Postnatal Blood Lead Exposure and Adrenocortical Responses to Acute Stress in Children

**DOI:** 10.1289/ehp.10391

**Published:** 2007-11-17

**Authors:** Brooks B. Gump, Paul Stewart, Jacki Reihman, Ed Lonky, Tom Darvill, Patrick J. Parsons, Douglas A. Granger

**Affiliations:** 1 Department of Psychology, State University of New York College at Oswego, Oswego, New York, USA; 2 Trace Elements Laboratory, Wadsworth Center, New York State Department of Health, Albany, New York, USA; 3 Department of Environmental Health Sciences, School of Public Health, The University at Albany, Albany, New York, USA; 4 Behavioral Endocrinology Laboratory, Department of Biobehavioral Health, Pennsylvania State University, State College, Pennsylvania, USA

**Keywords:** adrenocortical, children, cortisol, HPA axis, lead, metal pollution, Pb, stress

## Abstract

**Background:**

A few recent studies have demonstrated heightened hypothalamic–pituitary–adrenal (HPA) axis reactivity to acute stress in animals exposed to heavy metal contaminants, particularly lead. However, Pb-induced dysregulation of the HPA axis has not yet been studied in humans.

**Objective:**

In this study, we examined children’s cortisol response to acute stress (the glucocorticoid product of HPA activation) in relation to low-level prenatal and postnatal Pb exposure.

**Methods:**

Children’s prenatal blood Pb levels were determined from cord blood specimens, and postnatal lead levels were abstracted from pediatrician and state records. Children’s adrenocortical responses to an acute stressor were measured using assays of salivary cortisol before and after administration of a standard cold pressor task.

**Results:**

Pb exposure was not associated with initial salivary cortisol levels. After an acute stressor, however, increasing prenatal and postnatal blood Pb levels were independently associated with significantly heightened salivary cortisol responses.

**Conclusions:**

Our results suggest that relatively low prenatal and postnatal blood lead levels—notably those below the 10 μg/dL blood lead level identified by the Centers for Disease Control and Prevention for public health purposes—can alter children’s adrenocortical responses to acute stress. The behavioral and health consequences of this Pb-induced HPA dysregulation in children have yet to be determined.

Recent research has evaluated the effects of lead exposure on responses of the hypothalamic–pituitary–adrenal (HPA) axis to acute stress. These responses have been considered in animals, through assessment of glucocorticoid blood levels before and after the onset of an acute stressor. Several studies (Cory-Slechta et al. 2004; Virgolini et al. 1999, 2004) have demonstrated a significant positive association in rats between Pb exposure and baseline plasma corticosterone (the glucocorticoid equivalent of cortisol in humans). In addition, many studies have shown that greater Pb exposure is associated with heightened corticosterone reactivity to acute stress [Baos et al. 2006; Cory-Slechta et al. 2004; Virgolini et al. 2004 (in rats)]; however, in some cases, Pb exposure is associated with either a diminished glucocoticoid response to acute stress [Levesque et al. 2003 (in yellow perch); Virgolini et al. 2004 (in rats)] or no significant effect on acute stress reactivity (Virgolini et al. 2004). To our knowledge, the HPA response to acute stress as a function of Pb exposure has not been evaluated in humans. In the present study we consider salivary cortisol response to acute stress, in children with low-level Pb exposure.

Several studies have considered factors that affect children’s adrenocortical responses to acute stress. Such research has generally focused either on intrinsic differences such as temperament (van Bakel and Riksen-Walraven 2004) and attachment (Gunnar et al. 1989) or social contextual factors such as parental maltreatment (Cicchetti and Rogosch 2001) and abuse (De Bellis et al. 1994). Although exposure to environmental toxicants may co-vary with these variables (e.g., Gump et al. 2007), the study of toxicant effects is relatively novel to developmental psychobiology. Similarly, the study of acute stress responses in children is novel in neurotoxicology. Notably, however, we recently reported a positive association between blood Pb and cardiovascular reactivity to acute stress in children (Gump et al. 2005). Here we consider the further association of blood Pb to adrenocortical reactivity to acute stress in these children.

Pb-induced increases in HPA reactivity in children are likely to have far-reaching consequences. The association of trace metal concentrations in blood with cardiovascular disease has been assessed in various epidemiologic studies. Such studies suggest positive associations between Pb and blood pressure (Hu et al. 1996; Pirkle et al. 1985; Schwartz 1995), left ventricular hypertrophy (Schwartz 1991), and cardiovascular disease mortality (Voors et al. 1982). The mechanism explaining the associations between Pb and cardiovascular disease risk is not clear; however, it is presumed to involve a number of systems that regulate vasoconstriction and vasodilation, including the HPA system (Rogers et al. 2003; Virgolini et al. 2005). Therefore, it may be that Pb-induced increases in cardiovascular disease risk are produced partially by underlying Pb-induced increases in HPA responses to acute stress in children.

In addition to cardiovascular effects of Pb exposure, low-level Pb exposure is associated with cognitive deficits in children (Bellinger et al. 2003; Canfield et al. 2003). In some studies, HPA dysregulation and consequent hypercortisolemia have shown a significant negative association with cognitive functioning (McEwen 1998; Sapolsky 1996). Therefore, low-level Pb exposure may cause chronic elevations in cortisol levels and thereby produce cognitive deficits (cf. Cory-Slechta et al. 2004). The present research constitutes the first step in testing these potential pathways, by determining whether blood Pb levels alter adrenocortical responses to acute stress in children.

## Methods

### Participants

Participants were recruited in the context of an ongoing longitudinal study of the effects of environmental toxicants on development (Lonky et al. 1996; Stewart et al. 2000). Of the 202 children currently enrolled in the Oswego Children’s Study, we included 169 children (91 females and 78 males) in the present study. Some children were not included because they were either not tested with the stressor tasks (*n* = 25) or had missing salivary cortisol samples (*n* = 8). Reasons for not being tested included inability to schedule within the testing window (*n* = 16), technical problems (*n* = 4), and refusal (*n* = 5). Prenatal Pb levels were available for 154 children and postnatal Pb levels were available for 120 children. The child’s response to an acute laboratory stressor was assessed within 2 weeks of attaining 9.5 years of age, and the family was paid $60 for participation in the current visit.

### Acute psychological stressor

The child was asked to submerge his or her dominant arm in a 1-gal tub with one part ice to one part water, for 1 min. This “cold pressor” task is commonly used in protocols designed to test neuroendocrine reactions to acute stress (e.g., Kapuku et al. 2002; Treiber et al. 1990). Participants were informed of the time remaining during the minute, to encourage completion of the task, although the instructions for the task clearly informed the participants that they were free to withdraw the arm if it became too painful. As a further precaution, pain ratings (1 = not at all painful; 7 = extremely painful) were assessed on a visual-analogue scale every 10 sec, and participants were reminded they were free to remove the arm if and when they rated the experience as a “5” or higher. The amount of time for which the child kept the arm submerged (i.e., tolerance time) and the average pain rating during the task were recorded. The cold pressor task is known to reliably evoke an adrenocortical response, and was therefore always administered first in the series of acute stress tasks. Only 11.8% of children in our study refused this task, defined as a tolerance time < 20% of the full 1 min—or 12 sec. The remaining children tolerated the cold pressor for 40 sec, on average. Notably, this tolerance time was not significantly associated with prenatal or postnatal lead exposure (*p* > 0.50). After the cold pressor task, we administered two cognitive stressors, mirror tracing and reaction time tasks (in counterbalanced order). As previously reported (Gump et al. 2005), these stressors were designed to assess cardiovascular reactivity; they are not discussed further here.

### Procedure

On the day of testing, the participant arrived at the laboratory at about 1630 hours (mean ± SD, 1628 ± 1.36) and before beginning read and signed an assent form, while his or her parents read and signed a separate consent form approved by the Institutional Review Board of SUNY Oswego. The laboratory session began with measurements of the child’s height and weight. Each experimental session comprised the following: *a*) an initial rest period (10 min); *b*) a cold pressor task (1 min of submerging the dominant arm in ice water followed by a 2-min recovery); *c*) an intertask rest (8 min); *d*) a choice reaction time task (3 min); *e*) an intertask rest (8 min); *f* ) a mirror-tracing task (90 sec; 5 trials); and *g*) a final recovery/rest period (10 min).

### Blood Pb measurements

#### Prenatal

Cord blood specimens were collected at delivery into 3-mL lavender top (Na_2_EDTA) evacuated glass tubes. Over the course of the study, both Vacutainer (Becton-Dickinson, Franklin Lakes, NJ) and Monoject (Terumo Corp., Ann Arbor, MI) tubes were used. However, each lot of blood tubes and needles used, regardless of source, was prescreened for Pb contamination and was certified for blood Pb measurements in the study by the analyzing laboratory. Specimens were shipped to the New York State Department of Health’s Wadsworth Center and were analyzed for Pb and erythrocyte protoporphyrin, a biochemical marker of Pb exposure. Each specimen was analyzed for Pb in duplicate, using a method based on electrothermal atomic absorption spectrometry (ETAAS) with longitudinal Zeeman-effect background correction. The method has been fully validated and is described in detail elsewhere (Parsons and Slavin 1993). In summary, the method detection limit (MDL) is 1 μg/dL, and the repeatability—day-to-day precision—ranges from 1.2 to 3.5% at blood Pb concentrations around 10 μg/dL, and is typically < 2% above 20 μg/dL. At the mean blood Pb concentration measured in this study, the day-to-day SD ranges from 0.1 to 0.3 μg/dL. The method has been routinely cross-validated against inductively coupled plasma mass spectrometry, and traceability to SI units is assured through analysis of standard reference materials for blood Pb (Palmer et al. 2006). The Wadsworth Center’s blood Pb laboratory is the New York State reference laboratory for this assay, and is responsible for operating the New York State blood Pb proficiency testing program.

#### Postnatal

Postnatal blood Pb data were collected for the children at an average (± SD) age of 2.62 ± 1.20 years, through the children’s pediatricians and county public health agencies. New York State law requires blood Pb testing for all children before entering kindergarten, and only those laboratories certified by the New York State Department of Health’s Wadsworth Center can accept and analyze blood Pb specimens drawn in New York State. Although more than 75 laboratories are certified to perform blood Pb testing in New York State, blood specimens are typically sent to a variety of clinical laboratories in both the commercial and public health sectors, depending on local screening practices and health insurance coverage. Analytical techniques used for blood Pb are ETAAS and anodic stripping voltammetry (ASV). Specific laboratories used to analyze specimens from children enrolled in the Oswego Children’s Study included Quest Diagnostics Clinical Labs in Norristown, Pennsylvania, formerly SmithKline Beecham (32% of specimens, analyzed by ETAAS); Wadsworth Center, Albany, New York (30.7%, analyzed by ETAAS); Oswego Hospital Laboratory, Oswego, New York (20%, analyzed by ASV); A. Lee Memorial Hospital Laboratory, Fulton, New York (10.7%, analyzed by ASV); Quest Diagnostics Inc., Teterboro, New Jersey (4%, analyzed by ETAAS); and SUNY Upstate Medical University Clinical Pathology Laboratory, Syracuse, New York (3%, analyzed by ASV). These laboratories participate regularly in the New York State proficiency testing program that is organized by the Wadsworth Center and are certified under both New York State and federal regulations (Clinical Laboratories Improvement Act 1988). Blood Pb levels were determined on venous specimens for 79.9% of the reported data, with the remainder determined on capillary blood specimens. Although capillary blood Pb measurements can be compromised by contamination errors during collection, the Wadsworth Center has estimated that false positive errors, defined as a false positive proportion, are typically < 5% at 10 μg/dL (Parsons et al. 1997). The amount of background contamination observed in 95% of capillary specimens, though generally insignificant from a clinical perspective (< 1 μg/dL), does give rise to a larger relative error at concentrations approaching the MDL.

For blood Pb results that were reported as less than the MDL, we entered one-half the value of the reported MDL for the corresponding data points (cf. Schantz et al. 2001). For postnatal blood Pb, most children had either one (*n* = 76) or two (*n* = 35) blood Pb tests; however, a few children had three (*n* = 9) or more (*n* = 2) tests before 9 years of age. The median value was used for the children with more than one draw. Blood Pb levels in the Oswego cohort ranged from 1.5 to 13.10 μg/dL, with only six children having blood Pb concentrations > 10 μg/dL, the level defined by the Centers for Disease Control and Prevention (CDC) as elevated for public health purposes (CDC 1991).

### Cortisol assessment

To measure adrenocortical reactivity, we collected saliva specimens at four points during the stress protocol: during the baseline period, twice after the acute stress task (21 and 40 min after the start of the cold pressor task), and during recovery (60 min after the cold pressor task). Following Kivlighan and Granger (2006), participants were asked to imagine chewing a piece of their favorite food, while moving their jaws as if they were really chewing and to gently force the pooling saliva through a short plastic straw into a 5-mL cryovial. All specimens were immediately frozen at −20°C until transported on dry ice to Pennsylvania State University for cortisol assay.

On the day of testing, all specimens were centrifuged at 3,000 rpm for 15 min to remove mucins. Specimens were assayed for salivary cortisol using a highly sensitive enzyme immunoassay, cleared by the U.S. Food and Drug Administration (2007, section 510k) for use as an *in vitro* diagnostic measure of adrenal function (Salimetrics, State College, PA). The test used 25 μL saliva, had a lower limit of sensitivity of 0.007 μg/dL, a range of sensitivity from 0.007 to 1.8 μg/dL, and average intra-and interassay coefficients of variation of < 5% and 10%, respectively. All samples were assayed in duplicate, and with the average of the duplicates used for all test results. Cortisol units are expressed in micrograms per deciliter. To create a more normal distribution, we eliminated a single outlier (with a value that was > 3 SD above the mean for cortisol change at both 40 and 60 min) and used a logarithmic transformation (cf. Baos et al. 2006).

Cortisol levels follow a circadian cycle, with peak levels in the morning, a steady drop during the morning hours, and a relatively stable plateau in the afternoon to early evening. In addition, cortisol levels are affected by recent meals. Therefore, all subjects were scheduled to begin the protocol in the afternoon (approximately 1630 hours) and were given instructions to have no snacks during the 1 hr preceding the testing. If the child reported having had a recent snack, the session was either rescheduled or briefly delayed, depending on the timing of the snack. In addition, participants were instructed *a*) to avoid dairy products for 30 min before collections (restriction based on evidence that some bovine hormones cross-react in immunoassay), *b*) to rinse their mouths with water 10 min before sample collections (no snacks were provided between sample collections), and *c*) to not brush their teeth within 1 hr of testing, so as to avoid blood contamination in saliva (two participants were rescheduled due to injuries or surgery in the oral cavity within the preceding 48 hr (Kivlighan et al. 2004).

### Potential confounders

#### Psychosocial variables

To strengthen our inferences regarding children’s blood Pb levels, we considered various potential confounding variables, including characteristics measured during pregnancy, at birth, at 7 years of age, and at 9.5 years of age. Measures included paternal height and weight, maternal prepregnancy weight and height, maternal weight gain during pregnancy, maternal reported illness during pregnancy, obstetric complications (using the Ballard and a measure of optimality), head circumference at birth, birth weight, gestational age, maternal substance use during pregnancy (e.g., cigarettes, alcohol), the Home Observation for Measurement of the Environment (HOME), the Clinical Epidemiological Studies–Depression (CES-D; Radloff 1977) inventory, the Four Factor Index of Social Status (Hollingshead 1975) to measure socioeconomic status (SES), and body mass index (BMI; weight (kilograms)/height (meters)^2^). A complete list of these variables is provided in Table 1. Because of skewed distributions, the following measures were log-transformed (using a base 10 log-transformation) before use in any analysis: maternal illness (using an illness checklist); vitamin use during pregnancy (number/week); prescription-drug use during pregnancy (number/week); nonalcoholic substance use during pregnancy (specifically, herbal tea, decaffeinated coffee, diet soda, and decaffeinated soda in drinks/month); and alcohol use during pregnancy (drinks/day).

#### Other toxicants

We considered other potentially important prenatal toxicant exposures by assessing cord blood levels of polychlorinated biphenyls (PCBs), 1,1-dichoro-2,2-*bis*(*p*-chlorophenyl)ethylene (*p,p*′-DDE), and hexachlorobenzene. We assessed mercury levels from maternal hair cuttings, with position along hair strands used to differentiate mercury levels during the first and second halves of pregnancy. Further details on these other toxicant measures can be found in prior publications (Lonky et al. 1996; Stewart et al. 1999).

### Data analysis

#### Data reduction

We computed change scores for cortisol by subtracting initial levels from the task means. Therefore, our primary outcome variable was change in cortisol at 21, 40, and 60 min after the initiation of the cold pressor task.

#### Statistical treatment of potential confounders

A list of all the covariates considered in the present analysis, and their correlations with outcome measures, is shown in Table 1. The decision rules for the inclusion of covariates provide objective, comprehensive, and rigorous control for potential confounders. The method used in the current study is consistent with that employed previously by us (Darvill et al. 2000; Stewart et al. 2000, 2003, 2006) as well as by others (Jacobson and Jacobson 1996). Any potential confounding variables that were found to be even marginally related (*p* < 0.20) to cortisol responses served as covariates in all analyses. Several studies (Maldano and Greenland 1993; Mickey and Greenland 1989) have demonstrated that this alpha level (0.20) is effective in guarding against confounders in Monte Carlo simulations. Use of this criterion also allows major predictors of outcome to enter the equation, even if such variables are unrelated to exposure. Statistical power is thereby increased through reduction of the error term in the regression equation (Kleinbaum et al. 1988). We determined relationships between pairs of covariates and outcome through single-pass, bivariate correlations between each covariate and each outcome. Each cortisol assessment (in separate multiple regression equations) was regressed on these covariates and the residuals saved for use in subsequent analyses. The number of covariates in these equations varied from 16 to 6 (Table 1). This number of predictors relative to subjects is well within the 1:5 limits suggested by some (Tabachnick and Fidell 1989) and provides appropriate power for multiple regressions with medium effect sizes (Green 1991).

To further minimize residual confounding, we tested each covariate that failed to meet the *p* < 0.20 entry criterion, to see whether it affected the final outcome of the analysis. Monte Carlo simulations have empirically demonstrated that this additional change-in-estimate criterion, whereby a covariate is added to the equation if it changes the association (beta coefficient) between exposure and outcome by ≤ 10%, is an extremely effective and rigorous means of controlling residual bias in multivariate correlational data sets (Maldano and Greenland 1993; Mickey and Greenland 1989). We proceeded to include all covariates that even marginally (> 10%) altered the outcome, even if that outcome was already statistically significant. We are thus afforded strong assurance that no potentially important confounder was excluded. This approach has been used successfully in the Pb (Bellinger et al. 1992) and PCB (Stewart et al. 2005, 2006) literature.

Every analysis, therefore, included all covariates related to the outcome at *p* < 0.20, and any remaining covariates that even marginally changed the relationship between exposure and outcome (> 10% change in beta) of that analysis. Only relationships that were statistically significant after this two-tiered approach were considered meaningful.

#### Statistical treatment of predictor variables (blood Pb)

Although the distribution of pre-natal and postnatal blood Pb levels was nearly normal, regression models can be sensitive to the presence of outliers. In addition, use in the analyses of a value of one-half the MDL for postnatal blood Pb levels reported as below the MDL, resulted in differing Pb values, as a function of the testing laboratory’s MDL (either 3 or 5). Therefore, as was done previously (Gump et al. 2005), we focused our analyses on Pb quartiles but repeated these analyses with Pb data as a continuous variable (using SAS PROC REG; SAS Institute Inc., Cary, NC). For the analysis of quartiles, we included all participants who had nondetectable levels in the 1st quartile. For prenatal Pb exposure, quartiles corresponded to the following blood Pb levels: ≥ 1.0 (1st quartile; *n* = 37), 1.1–1.4 μg/dL (2nd quartile; *n* = 39), 1.5–1.9 μg/dL (3rd quartile; *n* = 36), and 2.0–6.3 μg/dL (4th quartile; *n* = 42). For postnatal Pb exposure, quartiles corresponded to the following blood Pb levels: 1.5–2.8 μg/dL (1st quartile; *n* = 28), 2.9–4.1 μg/dL (2nd quartile; *n* = 32), 4.2–5.4 μg/dL (3rd quartile; *n* = 29), and 5.5–13.1 μg/dL (4th quartile; *n* = 29). In the analyses of quartiles, we used SAS PROC GLM (SAS Institute Inc.) with a linear contrast to test the effects of increasing blood Pb levels controlling for all potential confounds (i.e., covariates).

## Results

### Potential confounders and salivary cortisol

Table 1 presents prenatal, perinatal, and other characteristics at the time of testing for the children and their mothers in our cohort. All children were tested within 2 weeks of attaining 9.5 years of age. Bivariate correlations are shown for each covariate across the four cortisol specimens.

**Figure 1:**
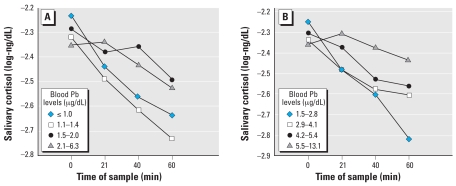
Children’s unadjusted initial salivary cortisol levels (log-ng/dL) and after an acute stress task as a function of quartiles of prenatal (*A*) and postnatal (*B*) Pb exposure.

As a preliminary analysis, we conducted a 4 (blood Pb quartile) × 4 (time of saliva sample) mixed factorial analysis of variance, with the saliva sample being a repeated measure. This analysis was initially performed for prenatal and postnatal Pb using a test for the linear contrast across quartiles, but without covariate control. These analyses revealed a significant interaction between the time of the cortisol sample and prenatal blood Pb levels, *F* (3, 411) = 4.79, *p* < 0.005, as well as postnatal blood Pb levels, *F* (3, 315) = 7.35, *p* < 0.0001. The mean values from these analyses are shown in Figure 1. After covariate adjustment, these interactions remained significant for prenatal [*F* (3, 393) = 3.19, *p* < 0.05] and postnatal [*F* (3, 264) = 2.90, *p* < 0.05] Pb exposure. To further analyze this interaction, we first considered initial adrenocortical levels as a function of Pb exposure.

### Initial adrenocortical levels and blood Pb exposure

After adjustment for covariates, pre-natal Pb quartiles were not significantly associated with initial cortisol levels, *F* (1, 142) = 0.01, *p* > 0.25. Similar results were obtained when prenatal Pb levels were analyzed as a continuous variable, *F* (1, 144) = 0.24, *p* > 0.25. After adjustment for covariates, post-natal Pb quartiles were not significantly associated with initial cortisol levels [*F* (1, 101) = 1.89, *p* > 0.15], nor were postnatal Pb levels when analyzed as a continuous variable [*F* (1, 103) = 1.70, *p* > 0.15]. Having established that initial levels of salivary cortisol were unaffected by prenatal and postnatal Pb exposure, we next analyzed changes from initial (i.e., reactivity) as a function of Pb exposure.

### Adrenocortical responses to acute stress and blood Pb exposure

**Figure 2:**
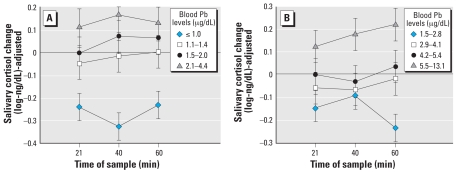
Children’s salivary cortisol reactivity (adjusted for covariates) as a function of prenatal (*A*) and postnatal (*B*) lead quartiles. Error bars represent SEs.

Prenatal Pb exposure had a significant effect on cortisol responses to acute stress at 9.5 years of age, with increasing Pb associated with an increasing cortisol response, *F* (1, 134) = 11.78, *p* < 0.001 (for linear contrast). This association was equivalent for samples collected at 21, 40, and 60 min [*F* (2, 268) = 0.28, *p* > 0.25 for the time × linear contrast interaction]. The adjusted means for this analysis are shown in Figure 2A. The effect of prenatal Pb exposure was somewhat stronger after additional adjustment for postnatal Pb levels, *F* (1, 87) = 18.20, *p* < 0.0001.

Postnatal Pb exposure had a significant effect on cortisol response to acute stress at 9.5 years of age, with increasing Pb associated with an increasing cortisol response, *F* (1, 98) = 11.21, *p* < 0.005. This association was again equivalent for samples collected at 21, 40, and 60 min [*F* (2, 196) = 1.89, *p* > 0.25, for the time × linear contrast interaction]. The adjusted means for this analysis are shown in Figure 2B. The effect of postnatal Pb exposure, although reduced, remained significant after additional adjustment for prenatal Pb levels, *F* (1, 83) = 4.06, *p* < 0.05.

**Figure 3:**
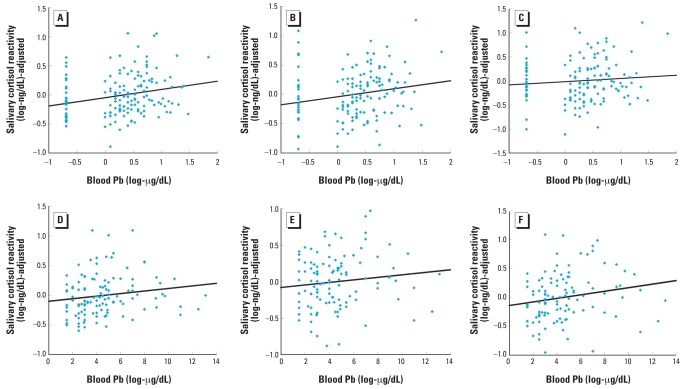
Children’s salivary cortisol reactivity (adjusted for covariates) at 21 (*A*, *D*) 40 (*B*, *E*), and 60 min (*C*, *F*) as a function of prenatal (*A–C*) and postnatal (*D–F*) lead exposure analyzed as continuous variables. Regression coefficients were β = 0.24, 0.24, 0.17, 0.25, 0.20, and 0.23 for *A–F*, respectively.

We next performed regression analyses, with covariates entered first, followed by Pb levels as a continuous variable. As shown in the upper row of Figure 3, prenatal Pb exposure was significantly associated with cortisol reactivity at 21 min [*t* (1, 147) = 2.97, *p* < 0.005], 40 min [*t* (1, 141) = 2.82, *p* < 0.01], and 60 min [*t* (1, 138) = 1.98, *p* < 0.05]. Similarly, postnatal Pb exposure was significantly or marginally significantly associated with cortisol reactivity at 21 min [*t* (1, 108) = 2.20, *p* < 0.05], 40 min [*t* (1, 103) = 1.84, *p* < 0.10], and 60 min [*t* (1, 102) = 2.31, *p* < 0.05], as shown in the lower row of Figure 3. Notably, PCB, mercury, and DDE levels were not significantly associated with cortisol levels in these children (*p* > 0.10).

**Figure 4:**
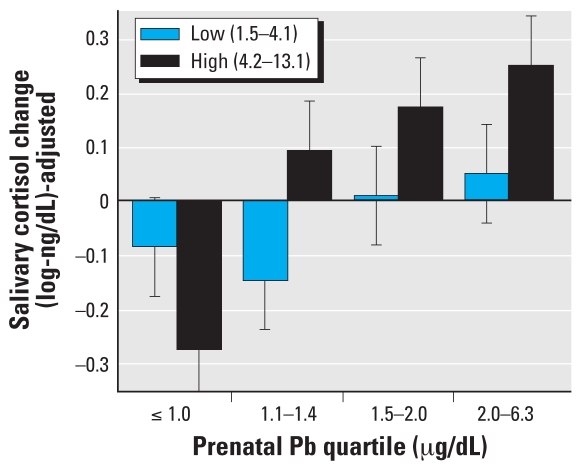
Combined effects of prenatal (as quartiles) and postnatal lead exposure (low vs. high) on children’s salivary cortisol reactivity (adjusted for covariates ± SE).

Finally, we tested SES and sex as potential effect modifiers, as found in previous research on Pb-induced changes in glucocorticosteroids (Cory-Slechta et al. 2004). Neither SES (measured at 1 and 9.5 years of age) nor sex significantly interacted with prenatal or postnatal Pb exposure effects (all *p* > 0.25). In addition, the interaction between Pb exposure timing (prenatal and postnatal) was not significant [*F* (9, 75) = 1.10, *p* > 0.25]; however, we do illustrate the additive nature of prenatal and postnatal Pb exposure on mean cortisol reactivity (adjusted for covariates) in Figure 4.

## Discussion

In the present study we examined the relationship between children’s prenatal and postnatal Pb exposures and subsequent adrenocortical activity at rest as well as in reaction to acute stress, measured at 9.5 years of age. We found that initial cortisol levels were not significantly associated with either prenatal or postnatal Pb exposure. This null association with basal glucocorticosteroid levels is consistent with findings in Pb-exposed birds (Baos et al. 2006), although studies in rats have shown increases in basal glucocorticoid levels in response to Pb exposure (Cory-Slechta et al. 2004; Vyskocil et al. 1990). For adrenocortical reactivity to acute stress, we found a significant positive association with both prenatal and postnatal Pb exposure. Moreover, although prenatal and postnatal Pb levels were positively correlated, their effects on cortisol reactivity were independent. Studies in animals have demonstrated a similar positive association between lead exposure and glucocorticoid reactivity (e.g., Baos et al. 2006; Cory-Slechta et al. 2004), but we are not aware of any studies that have yet demonstrated this association in humans.

The mechanism(s) for Pb effects on the HPA axis are not well understood. Pb exposure has been shown to affect the brain’s mesocorticolimbic dopaminergic system (Cory-Slechta et al. 1997) and could thereby potentiate HPA axis responses to stress (Cory-Slechta et al. 2004). Other possible mechanisms include direct effects on the hypothalamus (Bratton et al. 1994) or pituitary gland (Huseman et al. 1987), changes in brain catecholamine levels within the hypothalamus (Virgolini et al. 2005), or alterations to cytokine levels (Hemdan et al. 2005) and corresponding HPA feedback (Marx et al. 1998). Pb exposure could also indirectly affect cortisol levels via changes in body weight (Scavo et al. 1988) or nutrition (Giovannini et al. 1990) produced by Pb-induced mood changes (Lisboa et al. 2005).

A few concerns related to the current study should be noted. First, postnatal Pb was measured in various laboratories that used an MDL of either 3 or 5 μg/dL, whereas prenatal Pb was measured in just one laboratory using a single, well-characterized method (Wadsworth Center, NY) with an MDL of 1 μg/dL. Therefore, the effect of prenatal Pb exposure could have been stronger relative to postnatal Pb effects, because a more sensitive and precise measurement method (and corresponding reductions in measurement uncertainty) was used in a single laboratory for the prenatal exposure assessment.

The second concern is the consistent effect of Pb exposure across the 21-, 40-, and 60-min cortisol specimens. If Pb exposure affects only adrenocortical reactivity, and if 60 min is a sufficient length of time for recovery, then Pb exposure effects should have become attenuated by 60 min after the acute stress task. However, the effects of prenatal and postnatal Pb exposure on cortisol reactivity were not significantly different for the three cortisol samples. There are two possible explanations for this pattern of findings. Either 60 min was insufficient for the children’s recovery, or else our initial level did not truly represent resting adrenocortical activity. Although it is not uncommon for salivary cortisol changes to persist even after 60 min (e.g., Gruenewald et al. 2004), recovery is typically expected by 45–60 min (Kuhn 1989). With respect to the second possibility, establishment of a true adrenocortical baseline is unfortunately not straightforward. Studies have demonstrated a positive association between adrenocortical activity and exposure to a novel situation (al’Absi and Lovallo 1993; Fishman et al. 1962), although not all studies have corroborated this association (Hertsgaard et al. 1992). Our behavioral medicine protocol was quite novel for the children enrolled in the Oswego Children’s Study (a study primarily focused on cognitive development), so it is quite possible that the children’s salivary cortisol levels at “baseline” were already elevated in response to this unfamiliar situation. On the other hand, direct comparisons between home and laboratory cortisol levels in young children have shown lower cortisol levels in the laboratory at baseline, relative to levels in the home (Gunnar et al. 1989). Future testing should include home cortisol assessment, to determine whether the current protocol evokes differences from home levels. Such a study would help determine whether Pb affects HPA reactivity or HPA feedback sensitivity—an important distinction that would help direct future studies of Pb-induced HPA dysregulation. For example, if Pb exposure affects HPA feedback sensitivity, Pb interference with type I and II corticosteroid receptors or impaired glucocorticoid inhibition of adrenocorticotropic hormone secretion might be a target for future study.

That our assessment of postnatal Pb occurred approximately 7 years before our assessment of adrenocortical activity is a third concern. However, multiple measures of Pb over time have been shown in prospective studies of children to be highly correlated (e.g., Wasserman et al. 2000). Furthermore, any nonsystematic misclassification of postnatal Pb levels due to this gap between the two assessments would only underestimate the actual association between postnatal Pb levels and adrenocortical dysregulation.

### Conclusions

The present study demonstrated that prenatal Pb exposure was associated with a significant increase in the child’s cortisol reactivity to acute stress at 9.5 years of age. Comparable but independent effects on cortisol reactivity were observed for postnatal Pb exposure. Although the long-term implications for this association are unclear, they are undoubtedly complex. Increased HPA axis activity is associated with a number of emotional, behavioral, and physical problems in children (Charmandari et al. 2003). However, chronic hyperactivity of the HPA axis (e.g., through chronic Pb exposure) could lead to hypocortisolemia via reduced hormone bioavailability and/or reduced sensitivity (Raison and Miller 2003). Hypocortisolemia is associated with a number of increased risks, including post-traumatic stress disorder (Delahanty et al. 2000; Yehuda et al. 1995a, 1995b), substance abuse (Moss et al. 1999), and physical disease (Heim et al. 2000). Therefore, it will be important to continue to follow these children, and to determine the consequences of these early effects of Pb exposure on glucocorticoid reactivity. Furthermore, future studies should consider cortisol reactivity as a potential mediator for disorders that can be induced by Pb, such as fatigue (Lilis et al. 1985), cognitive impairment (Canfield et al. 2003), and immune disorders (Dietert et al. 2002). Finally, the effects that we found in the study were significant in a population with low levels of Pb exposure, levels well below the 10 μg/dL defined by the CDC as elevated in young children.

## Figures and Tables

**Table 1:** Relationships between covariates and salivary cortisol levels initially and after the onset of the acute stressor.

	Time after the onset of acute stress (min)			
Covariate	Initial level	21	40	60
Demographic				
Maternal education	0.075	−0.028	−0.015	−0.049
Paternal education	0.051	−0.030	0.045	−0.036
Parity of child	0.162**	0.014	0.049	0.068
SES score (1 year)	−0.012	0.101*	0.012	0.009
SES score (9 years)	0.018	−0.137*	−0.086	−0.122*
Maternal IQ	−0.020	−0.035	−0.002	0.017
Maternal sustained attention (CPT)	−0.045	−0.175**	−0.136*	−0.057
Maternal impulsive responding (CPT)	−0.059	0.078	−0.021	0.030
Maternal depression (past)	0.034	0.106*	0.196*	0.188**
Maternal depression (current)	−0.058	0.196**	0.147*	0.157*
Maternal age	0.071	−0.092	0.007	0.004
Maternal height	−0.029	0.046	0.094	0.105*
Paternal age	0.209#	−0.181*	−0.025	−0.033
Paternal height	−0.029	0.046	0.094	0.105*
Paternal weight	0.055	0.018	0.048	−0.027
HOME 1 year	−0.148**	−0.096	−0.016	0.063
HOME 4.5 years	0.018	−0.236**	−0.119	−0.082
HOME 7 years	−0.054	−0.159**	−0.097	−0.087
No. of years at same address	−0.116*	−0.077	−0.011	0.001
No. of years near Great lakes	0.067	−0.040	0.031	0.033
Marital status	−0.072	0.063	−0.041	−0.012
Child care	−0.010	−0.070	−0.052	−0.026
Home care	−0.165**	0.086	−0.103*	−0.061
Health/nutrition				
Prepregnancy weight	−0.112*	0.035	0.081	0.087
Weight gain during pregnancy	−0.006	0.095	0.072	0.047
Stress before pregnancy	−0.006	0.010	−0.013	−0.015
Stress: 1st half pregnancy	−0.166**	−0.082	−0.082	−0.024
Stress: 2nd half pregnancy	−0.063	0.049	0.096	0.104*
Maternal illness history	−0.139*	0.029	0.068	0.140*
Obstetric optimality	−0.038	−0.013	0.015	−0.024
Vitamins during pregnancy	0.002	−0.022	−0.023	−0.006
Prescription meds. during pregnancy	0.032	0.034	0.055	−0.005
Nonprescription meds. pregnancy	0.012	0.040	−0.003	−0.006
Nutrition scale	0.025	0.047	−0.030	0.010
Child’s body mass index (age 9)	−0.146**	−0.039	−0.068	−0.046
Infant/birth characteristics				
Child sex	−0.039	0.093	0.211#	0.227#
Birth weight (g)	−0.145*	−0.061	0.006	0.078
Head circumference	−0.143*	−0.056	−0.017	0.031
Ballard: neuromuscular	−0.078	−0.155**	−0.033	−0.049
Ballard: physical	−0.015	−0.139*	−0.062	0.016
Gestational age at birth	−0.133*	−0.151*	−0.052	0.001
Substance use				
Cigarettes/day	0.085	−0.012	−0.054	−0.010
Secondhand smoke (hr/day)	−0.116*	0.048	−0.026	−0.006
Alcohol (no. drinks/day)	−0.062	0.033	0.007	0.068
Herbal tea (drinks/month)	−0.141*	−0.030	−0.105*	−0.114*
Decaffeinated coffee (drinks/month)	−0.111*	−0.049	−0.046	−0.045
Diet soda (drinks/month)	−0.117*	−0.049	−0.046	−0.045
Decaffeinated soda (drinks/month)	−0.044	0.132*	0.012	0.063
Caffeine beverages (drinks/month)	0.055	0.040	0.050	0.076

* *p* < 0.20,

** *p* < 0.05,

# *p* < 0.01
